# Impact of an online guided physical activity training on cognition and gut-brain axis interactions in older adults: protocol of a randomized controlled trial

**DOI:** 10.3389/fnagi.2023.1254194

**Published:** 2023-09-14

**Authors:** Simon J. Schrenk, Stefan Brodoehl, Stefano Flor, Christiane Frahm, Christian Gaser, Rami Abou Hamdan, Marco Herbsleb, Christoph Kaleta, Fabian Kattlun, Hans-Josef Müller, Christian Puta, Monique Radscheidt, Adriana L. Ruiz-Rizzo, Tannaz Saraei, André Scherag, Thomas Steidten, Otto W. Witte, Kathrin Finke

**Affiliations:** ^1^Department of Neurology, Jena University Hospital – Friedrich Schiller University of Jena, Jena, Germany; ^2^Institute of Experimental Medicine, Christian-Albrechts-University zu Kiel, Kiel, Germany; ^3^Department of Psychiatry and Psychotherapy, Jena University Hospital – Friedrich Schiller University of Jena, Jena, Germany; ^4^Department of Sports Medicine and Health Promotion, Friedrich-Schiller-University Jena, Jena, Germany; ^5^Department of Psychosomatic Medicine and Psychotherapy, Jena University Hospital – Friedrich Schiller University of Jena, Jena, Germany; ^6^Center for Sepsis Control and Care (CSCC), Jena University Hospital – Friedrich Schiller University of Jena, Jena, Germany; ^7^Center for Interdisciplinary Prevention of Diseases Related to Professional Activities, Friedrich-Schiller-University Jena, Jena, Germany; ^8^Center for Clinical Studies, Jena University Hospital – Friedrich Schiller University of Jena, Jena, Germany; ^9^Institute of Medical Statistics, Computer and Data Sciences, Jena University Hospital – Friedrich Schiller University of Jena, Jena, Germany

**Keywords:** cognition, physical activity, online intervention, fMRI, healthy, older adults, microbiome

## Abstract

**Introduction:**

By 2050, the worldwide percentage of people 65 years and older is assumed to have doubled compared to current numbers. Therefore, finding ways of promoting healthy (cognitive) aging is crucial. Physical activity is considered an effective approach to counteract not only physical but also cognitive decline. However, the underlying mechanisms that drive the benefits of regular physical activity on cognitive function are not fully understood. This randomized controlled trial aims to analyze the effect of an eight-week standardized physical activity training program in older humans on cognitive, brain, and gut-barrier function as well as the relationship between the resulting changes.

**Methods and analysis:**

One-hundred healthy participants aged 60 to 75 years will be recruited. First, participants will undergo an extensive baseline assessment consisting of neurocognitive tests, functional and structural brain imaging, physical fitness tests, and gut-microbiome profiling. Next, participants will be randomized into either a multi-component physical activity group (experimental condition) or a relaxation group (active control condition), with each training lasting 8 weeks and including an equal number and duration of exercises. The whole intervention will be online-based, i.e., participants will find their intervention schedule and all materials needed on the study website. After the intervention phase, participants will have their post-intervention assessment, which consists of the same measures and tests as the baseline assessment. The primary outcome of this study is the change in the cognitive parameter of visual processing speed from baseline to post-measurement, which will on average take place 10 weeks after the randomization. Secondary outcomes related to cognitive, brain, and microbiome data will be analyzed exploratory.

**Clinical trial registration:** https://drks.de/search/de/trial/DRKS00028022

## Introduction

Life expectancy is steadily increasing over the last decades (United Nations, 2022). With rising age, the risk of cognitive decline increases, too (Deary et al., 2009). Therefore, finding ways of preserving cognitive health in aging individuals is crucial, not only on an individual level but also concerning the health care system (Patterson, 2018).

There is mounting evidence that modifiable risk factors that can be addressed by daily habits and routines influence the individual course of cognition at an older age. The Lancet Commission on dementia prevention lists 12 relevant lifestyle-related changes that could potentially prevent around 40% of dementia cases (Livingston et al., 2020). Especially higher physical activity levels have been reported to be related not only to bodily health but also to better cognition in the elderly (Etnier et al., 1997; Erickson and Kramer, 2008; Gomez-Pinilla and Hillman, 2013) and lower risk of dementia in later life, in several cross-sectional studies (Brown et al., 2012; de Bruijn et al., 2013). Furthermore, a number of randomized controlled intervention trials (RCTs) in healthy older participants showed beneficial effects of physical interventions on cognitive measures indicating a causal link between increased physical and neurocognitive functioning (for reviews see Colcombe and Kramer, 2003; Smith et al., 2010; Gajewski and Falkenstein, 2016). The main benefits across these studies seem to be found in attentional and executive control processes, including processing speed and memory functions. However, a recent umbrella review by Ciria et al. (2023) implies that the empirical evidence for beneficial effects of exercise on cognition might be less conclusive and reliable than suggested in these reviews.

Prior studies attempting to analyze the mechanisms that might underlie the link between better physical fitness and cognition in older adults following exercise interventions showed enhanced cerebral blood flow (Barnes, 2015), volume changes in brain regions (Colcombe et al., 2006; Reiter et al., 2015), such as the frontal lobes, associated with attentional and memory processes, and functional connectivity changes (Voss et al., 2010) to be related to enhanced cognitive performance (Erickson et al., 2011; Chapman et al., 2013; Chieffi et al., 2017).

As, furthermore, evidence for the relevance of the integrity of the gut-brain axis as a bidirectional communication pathway between the gut microbiome and the brain is mounting (Rhee et al., 2009; Vogt et al., 2017; Cryan et al., 2019), the gut microbiome composition could also be a relevant factor underlying the effects of physical interventions on neurocognitive functions. The gut microbiome is the entity of microorganisms colonizing the gastrointestinal tract consisting of over 100 trillion microbes (Thursby and Juge, 2017). Its composition has a high interindividual variability among healthy individuals and higher microbial diversity is associated with better health status and immune reactions (Monda et al., 2017). Aging is known to be associated with decreased microbial diversity and decreased number of beneficial microbes (Woodmansey, 2007; Badal et al., 2020). These changes can be linked to higher susceptibility to infections and the development of neurodegenerative diseases like Alzheimer’s disease (Askarova et al., 2020; Bosco and Noti, 2021). Notably, a growing body of research in mice and humans shows that exercise can lead to a healthier microbiome by enhancing the number of beneficial microbes, resulting in positive effects on general health status and cognition (for reviews see Monda et al., 2017; Mailing et al., 2019). However, in humans, no RCTs on the question of whether exercise interventions influence the microbiome have been carried out so far. Previous studies were either only cross-sectional (Clarke et al., 2014), only assessed the impact of exercise in one group before and after an intervention (Munukka et al., 2018), or compared groups with different characteristics (Allen et al., 2018). These studies suggest that in young and middle-aged subjects, and in one non-randomized study also in older individuals (Morita et al., 2019), the human microbiome can be positively influenced by exercise. Addressing this impact with an RCT in older humans is therefore highly needed.

Moreover, to the best of our knowledge, it has not been established yet whether and how the different effects of physical exercise on cognition, on the gut microbiome, and on the brain are related to each other. Therefore, in our study, we will investigate the impact of a physical activity intervention on cognition, brain structure and function, the gut microbiome, and physical fitness in older humans in an RCT.

Based on prior research, we expect that participants randomly assigned to the physical activity intervention group will show higher improvements in the cognitive outcome measures. We further hypothesize that these exercise-induced changes in cognition are associated with changes in brain structure and function, as well as alterations in the gut microbiome composition.

## Methods and analysis

### Study design

This study is a monocentric, single-blinded, randomized, actively controlled intervention study with two arms. Participants are not informed about the underlying hypotheses of the study. An active control group is included to identify the impact of a physical activity intervention on the above-mentioned measures and to control for unspecific effects in terms of placebo-effects due to the occupation with a potentially health-promoting intervention or due to repetition of the test procedures. We use stratified randomization (stratum = sex) to allocate participants into either a specific intervention group (physical activity intervention) or an active control group (nonaerobic relaxation units). The study is conducted in Jena with pre-and post-assessments taking place at the Jena University Hospital and the Friedrich-Schiller-University Jena and the intervention taking place self-dependently at the participants’ homes.

### Sample size

The sample size calculation focused on our primary outcome, VPS (parameter *C*), derived from modeling the accuracy of the report in the whole report based on the TVA (Bundesen, 1990). Previously, an improvement in VPS *C* was achieved through alertness training (Penning et al., 2021). Based on the estimated effects of the study by Penning and colleagues, we expect a change in VPS of 3 letters per second (parameter *C*) with the standard deviation ranging between 4.7 and 6.4. To detect a mean difference of 3 letters per second (parameter *C*) with a standard deviation of 5 with 80% power and a two-sided significance level of α = 0.05 (power.t.test in R 4.0.5), 45 participants are needed for each study arm. To compensate for a slightly more complex analysis model and possible drop-outs, we will include five additional participants per arm whereby the total sample size adds up to 2 × 50 participants.

### Sample characteristics and recruitment

The inclusion and exclusion criteria for this study are listed in detail in Table 1. In brief, inclusion criteria comprise being at an age of 60 to 75 years, possessing a smartphone, having basic immunization against COVID-19 and being overall healthy. Exclusion criteria include specific non-communicable diseases, that affect the gut microbiome (e.g., diabetes, chronic obstipation, or diarrhea), that affect the ability to do sports or pose a risk when doing sports (e.g., stadium 3 and 4 cardiac diseases, and asthma), or that can affect cognition (neurological or psychiatric diseases). Intake of specific medication that might affect cognition or the gut microbiome composition lead to an exclusion, too. To ensure a risk-free MRI examination, contraindications for MRI are exclusion criteria as well (e.g., cardiac pacemaker and claustrophobia). A conspicuous value (≤86) in the cognitive screening test Addenbrooke’s Cognitive Examination III (ACE-III; Noone, 2015) is another exclusion criterion in order to exclude participants with signs of mild cognitive impairment and, thus, risk for a neurodegenerative process. Most exclusion criteria are checked in an interview before potential participants will be recruited. The cognitive status is assessed during the pre-assessment. Furthermore, a physician checks every participant before the self-monitored intervention to preclude any cardiovascular risks.

**Table 1:** Exclusion criteria for participants.

Relevant, uncorrected vision disorder
Evidence of cognitive impairment (ACE-III score of ≤86)
Relevant psychiatric or neurological pre-existing condition (e.g., major depression, focal damage of the brain due to operation or stroke, epilepsy)
Contraindications for MRI (e.g., cardiac pacemaker, non-removable metal parts in/at the body, claustrophobia)
Permanent medication with sedating substances
Loss of capacity of consent
Relevant, physical impairment
Stadium 3 and 4 cardiac diseases
Relevant systemic disease: cancer, severe kidney or liver disease, type 1 or 2 diabetes, diseases with intrinsic inflammation (e.g., arthritis, Crohn’s disease, asthma, chronic infections)
Treatments that influence glucose or microbiome composition: antidiabetics, antibiotics, immune suppressants, etc.
Chronic obstipation or diarrhea
Symptoms of clinical infection during the last month
Excessive alcohol/drug consumption
Severe eating disorder
Known disruption of iron balance
Chronic treatment with steroidal anti-inflammatory drugs
Treatment with proton pump inhibitors

We use different methods for recruiting participants for this study. First, flyers are distributed at selected locations in Jena. Furthermore, recruitment calls are printed in local newspapers and short features are broadcasted on local radio stations. Interested participants can contact the study team via email or telephone and are invited after inclusion and exclusion criteria were checked.

### Outcome measures

Primarily, we aim at assessing the effect on cognitive functions measured by various standardized and non-standardized cognitive tests. As we are only including cognitively healthy participants, reaching a ceiling effect in some of the established clinical tests designed for measuring cognitive impairment in older individuals or patients with brain dysfunction might be a pitfall. Therefore, our primary outcome is visual processing speed (VPS) which is derived from an experimental psychophysical whole report task based on the theory of visual attention (TVA; Bundesen, 1990). By computational modeling, a person’s individual VPS, in numbers of objects processed per second, can be estimated. We will focus on VPS because it has been shown to decrease with age (McAvinue et al., 2012; Ruiz-Rizzo et al., 2019), it is especially affected in individuals at risk for dementia (Ruiz-Rizzo et al., 2017), and it has been shown to improve after an experimental intervention in healthy, older individuals (Penning et al., 2021). Regarding the procedure, firstly, we will test whether an eight-week physical fitness intervention increases VPS in healthy older adults, based on the results of a previous study by Penning et al. (2021).

Secondly, to explore the underlying mechanisms of the impact of physical activity on healthy cognitive aging we will test whether the changes in the cognitive outcome measures are related to changes in brain structure and function, and the gut microbiome composition.

The primary outcome of this study is the change from pre-to post-assessment of the visual processing speed *C* (in elements/s), derived from the whole report based on the TVA (Bundesen, 1990).

The secondary outcomes will be derived from five different data categories: Data obtained from cognitive tests, psychological questionnaires, Magnetic Resonance Brain imaging (MRI), gut microbiome samples, and physical fitness assessments. See Table 2 for detailed descriptions of outcome measures and scheduling of data collection.

**Table 2:** Outcome measures.

Variables	Instruments	Pre- and post-assessment
Primary Outcome: Visual processing speed *C*	Whole report task (TVA)	NPT session
Visual short-term memory capacity *K*	Whole report task (TVA)	NPT session
Visual threshold *t_0_*	Whole report task (TVA)	NPT session
Top-down control *α*	Partial report task (TVA)	NPT session
Cognitive speed	Trail-Making-Test Part A	NPT session
Cognitive flexibility	Trail-Making-Test Part B	NPT session
Verbal memory	Verbaler Lern-und Merkfähigkeitstest (German version of the Auditory Verbal Learning Test)	Sports session
Visual memory	Rey-Osterrieth Complex Figure Test (ROCF)	Sports session
Inhibition ability	Farbe-Wort-Interferenz-Test (German version of the Color-Word-Interference-Test)	Sports session
Maximum oxygen uptake (VO_2_max)	Questionnaire, 6-min walking test, ergometer test	Sports session
Handgrip strength	Jamar^®^ Smart digital hand dynamometer (Performance Health, Warrenville, IL),	Sports session
BrainAGE	Structural brain imaging data (MRI)	MRI session
Functional brain connectivity	Functional brain imaging data (fMRI)	MRI session
Structural brain connectivity	Diffusion-tensor brain imaging data (DW-MRI)	MRI session
Sequencing data	16S sequencing, optionally metagenomics	NPT / Sports / MRI session
Bacterial community composition	16S sequencing, optionally metagenomics	NPT / Sports / MRI session
Diversity indices	16S sequencing, optionally metagenomics	NPT / Sports / MRI session
Diversity indices	16S sequencing, optionally metagenomics	NPT / Sports / MRI session
Fecal metabolomics data (optionally for a subset of samples)	16S sequencing, optionally metagenomics	NPT / Sports / MRI session

TVA, Theory of Visual Attention; NPT, Neuropsychological Testing; (f)MRI, (functional) Magnetic Resonance Imaging.

The pre-and post-assessments comprise a multitude of different tests and measurements which are conducted at three different sessions at different facilities. The majority of neuropsychological tests (NPT) are conducted in an NPT lab at Jena University Hospital (JUH). The physical fitness tests take place in a lab at the sports medicine department of Friedrich Schiller University Jena. Furthermore, the remaining neuropsychological tests are applied, and the online intervention is introduced in the sports medicine department. Lastly, brain imaging is done at the research MRI scanner of the JUH. For analysis of the gut microbiome composition, a stool-sample tube is collected at one of the appointments.

Based on the structural brain images, the individual BrainAGE score will be calculated. Machine learning approaches are used to derive the BrainAGE score, a morphometric parameter that is derived from multivariate, voxel-wise analyzes (Franke and Gaser, 2019).

### Cognitive data acquisition

Due to the length of all cognitive tests combined, the acquisition of cognitive data is split into two sessions during the pre-and post-assessments, respectively. The primary outcome VPS (parameter *C*) is derived from the non-standardized whole report task based on the TVA (Bundesen, 1990). This task is computer-based and conducted in a dimly lit room. To control for visual limitations, all participants first complete the MARS contrast sensitivity test. Following this, they complete a whole-report and then a partial-report paradigm within about 70 min. All trials are presented on a PC monitor (Asus 24-inch monitor, 1980 × 1,080 pixel screen resolution, 100 Hz refresh rate) with a black background. Participants see a central fixation circle followed 100 ms later by briefly presented letters [drawn from a set of 23 letters (ABCDEFGHJKLMNOPRSTUVWXZ)]. In a pretest phase, each participant’s individual exposure durations are determined. Participants report verbally, in any order and without time pressure, all letters they recognized (whole report) or letters of a pre-specified target color (partial report). The experimenter records the letters and starts the next trial.

**Figure 1:**
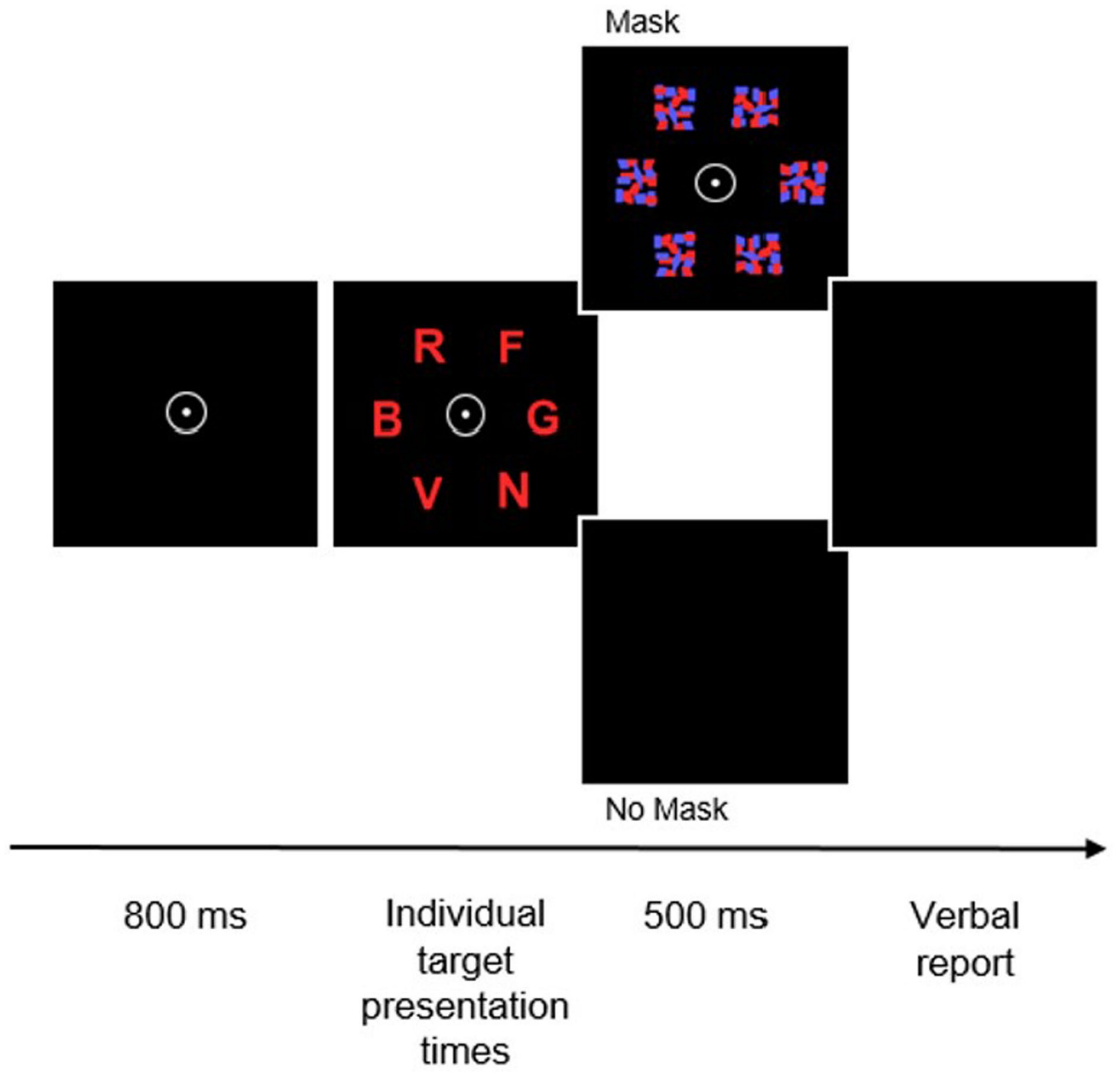
Trial sequence in the TVA-based whole-report task. After the presentation of a central fixation point for 800 ms, six random letters from a prespecified set are simultaneously presented for predetermined individual presentation times. Following that, presented stimuli are either masked or remain unmasked for 500 ms before participants are asked to verbally report the letters.

In the whole report, six equidistant red or blue letters are presented at five different exposure durations and arranged in a circle (see Figure 1 for a visual display of one trial). The task is to report as many letters as possible. An auditory cue (an 80 dB tone) is administered 200 ms before half of the trials. Furthermore, in half of the trials letter presentation is followed by visual masks on the prior position of the letter, to stop visual encoding. The whole report consists of four test blocks consisting of 84 trials each. Besides the primary outcome VPS *C*, the working memory storage capacity (in number of elements), and the visual perceptual threshold *t_0_* can be yielded.

In the partial report, a (red) single target letter, a target plus a (blue) distractor letter, or two (red) targets appear at the corners of an imaginary square located 7.5 cm around the fixation point. All stimuli are masked and there are no auditory cues. Participants have to report the target letter while ignoring distractors. This task also starts with a pretest phase in which exposure duration is determined and is followed by six test blocks with 48 trials each. Conduction of the partial report allows the estimation of the attentional-selectivity parameter top-down control *α*. Just like the whole report, the partial report is also not standardized.

All of the following neuropsychological tests are validated, standardized, and normed. Either we are using tests that were published and validated in German speaking countries or the officially translated versions of the validated original tests.

The Trail-Making-Test (TMT) is part of the neuropsychological test battery CERAD plus (Thalmann and Monsch, 1997). Part A is conducted to test for processing speed and part B to test for task switching. In the TMT-A, participants have to connect numbered circles with a pencil as fast as possible. In part B, which measures task-switching capabilities, circles with numbers and letters are presented and participants have to connect the circles, alternating between numbers and letters, in ascending and alphabetical order, respectively.

In addition, a virtual-reality-based (VR) adaption similar to the Trail-Making-Test A & B will be applied. Wearing VR-glasses with an integrated eye tracker, participants complete this test by fixating the targets without being limited by any potential manual motor speed changes potentially induced by aging. Again, the task will be to connect numbered circles presented in the visual field (part A) or to connect numbers and letters in alternating order (part B). The correctly fixated circles are highlighted on the screen. The VR-task is not yet validated. Therefore, the impact of the training on the data obtained with this test will only be analyzed in an exploratory manner. As it is unclear yet how visual problems might influence, the MARS contrast sensitivity test will be used to control for contrast sensitivity problems.

The “Verbaler Lern-und Merkfähigkeitstest” (VLMT; German version of the Auditory Verbal Learning Test; Helmstaedter et al., 2001) assesses different parameters of declarative verbal memory, such as learning, free delayed recall, and recognition. The instructor reads a list of 15 words from which as many as possible then have to be repeated by the participants. This procedure is repeated five times. After that, a list with 15 distractor words is read to the participant. Again, as many words as possible have to be repeated. Following the distractor list, participants have to recall as many words from the original list. A 30-min delay follows before participants have to freely repeat the words from the first list again. At last, the instructor reads a word recognition list consisting of the 30 words from both lists already heard by the participant as well as 20 semantic or phonetic distractor words. The participants have to decide whether or not a word belongs to the original list. For the pre-and post-assessment, two parallel test forms are used.

Using the Taylor Scoring System, the Rey-Osterrieth Complex Figure Test (ROCF; Strauss et al., 2006) is used to assess visuospatial ability and incidental memory. In this test, participants first have to copy a complex figure on a piece of paper. Following this, they get a blank paper and have to draw the figure again from memory. After a delay of 30 min, participants again have to draw the figure from memory. For the pre-and post-assessment, two parallel test forms are used.

The last cognitive test is the Farbe-Wort-Interferenz-Test [FWIT, validated German version of the Color-Word-Interference-Test (according to Stroop), Bäumler, 1985]. In the first test condition, participants have to read a list of color words. In the second condition, participants are presented colored bars of which they have to name the respective color. In the third and last condition, participants see color words that are printed in a different color than what the word says (e.g., the word “green” printed in red color). Their task is to name the color of the word while inhibiting the dominant response to reading out the word. In all three conditions, participants have to work as fast and correct as possible. Time is stopped for each of the three test runs.

The Addenbrooke’s Cognitive Examination III (Noone, 2015; Weiss et al., 2020) is only conducted in the pre-assessment, and serves as a screening tool to detect participants with initial cognitive impairment, in which case, participants are excluded from the study and referred to the Memory Center of JUH.

### Assessment of potential moderator variables

At the beginning of the study, demographic data like age, sex, and level of education are documented. Furthermore, the (German version of the) Multiple-Choice Vocabulary Intelligence Test (MWT-B; Lehrl, 1999) measures crystallized intelligence, a sociodemographic measure that is not expected to change after the intervention. To control for acute psychological conditions during the pre-and post-assessments as well as general personality traits, questionnaires are filled out. These include the (German versions of the) Beck Depression Inventory Revision (BDI-II; Beck et al., 1996), the Big-Five-Inventory (BFI-10; Rammstedt et al., 2013), the measurement of everyday cognition (ECog; Farias et al., 2008), the Hospital Anxiety and Depression Scale (HADS-D; Herrmann-Lingen et al., 2018), the Memory Functioning Questionnaire (MFQ; Gilewski et al., 1990), and the Patient Health Questionnaire (PHQ-9; Gräfe et al., 2004). The questionnaires are normed, standardized, and validated German versions of established questionnaires. The only exceptions are the ECog and the MFQ, for which no official German version was available and which we translated for this study. Data from these questionnaires will be used in an exploratory manner.

### MRI data acquisition

All assessments take place on a 3.0-T MR scanner (Trio, Siemens, Erlangen, Germany) to obtain echo-planar T2*-weighted image volumes (EPI) and transaxial T1-weighted structural images. Functional resting state data are acquired in one EPI session of 250 volumes. The first three volumes will be subsequently discarded due to equilibration effects. A functional-image volume comprises 100 transaxial slices including the whole cerebrum and cerebellum (voxel size = 1.4 mm × 1.4 mm × 1.4 mm, repetition time, TR = 1950 ms, echo time, TE = 33.6 ms).

T1-weighted anatomical 3D images are collected with the same MR scanner. The following scan parameters are used: TR = 2.25 s, TE = 3.03 ms, inversion time, TI = 900 ms, field of view, FoV = 256 × 256 mm2, flip angle = 9°, voxel resolution = 1 × 1 × 1 mm^3^, and 176 axial slices.

For Diffusion Tensor Imaging, the following parameters are used: TR 3318 ms, TE 87.40 ms, FoV 210 mm, flip angle 78°, voxel size 1.5 × 1.5 × 1.5 mm^3^, slices 96, first anterior to posterior, then vice versa.

### Microbiome data acquisition

At the first appointment of the pre-and the post-assessment, a stool collection tube (INVITEK Stool Collection Tubes with DNA Stabilizer, #1038111200, Berlin, Germany) is handed to the participants together with a stool collector (MED + ORG Stuhlauffanghilfe, Niedereschach, Germany). Participants have to collect the fecal sample at home according to the enclosed instructions and return the tube on one of the next pre-assessment appointments. They are advised to collect the post-assessment stool sample at an equal time of the day as the pre-sample in order to keep the two samples comparable. Furthermore, samples should be collected as close as possible to the day they are handing the sample in, even though the DNA stabilizer conserves the sample for up to 3 months at room temperature. Stool samples are then taken to the Integrated Biobank Jena of the JUH and frozen at −80°C.

### Sports data acquisition

At first, general anthropometric measures (body mass, height, hip and waist circumferences) as well as assessments of heart rate and heart rate variability are taken during resting conditions. After that, there are three approaches used to estimate the cardiorespiratory fitness (CRF) of a participant.

First, after an introduction to Borg’s scale of perceived exertion (RPE 6–20 scale; Borg, 1970), participants undergo a submaximal bicycle ergometer test, according to the test procedure after Ekblom-Bak et al. (2014; Björkman et al., 2016), in order to estimate their maximum oxygen uptake (VO_2_max). The test is performed on an electronically braked cycle-ergometer (ergoselect 100^®^, Ergoline, Bitz, Germany) in a climate-controlled laboratory environment. Participants are instructed to pedal at a cadence of 60 to 65 RPM and not to speak or adjust the position for the duration of the test. The total duration of the test is 8 min, with the initial 4 min performed at a low fixed power output of 30 watts, directly followed by 4 min at a higher individualized power output (aiming at an RPE of ≈14 on the Borg 6–20 scale). RPE is inquired in the second, third, and fourth minute of the two exercise stages. The heart rate of the participants is constantly measured throughout the test and capillary lactate blood samples are taken at rest, in the last minute of the first and second stages, as well as one and 3 mins after cessation of the test. Using sex-specific prediction equations (Björkman et al., 2016), VO_2_max can be predicted based on the independent variables age, change in HR per unit, change in power output, the difference in work rates, and HR at standard work rate. This submaximal cycle ergometer test and the underlying prediction model were developed on the basis of a graded maximal treadmill test. The adjusted *R*^2^ for this model are 0.81 (SEE = 0.31 L min^−1^) for ages between 50 and 64, and 0.81 (SEE =0.25 L min^−1^) for people older than 65 years. While measuring VO_2_max using maximal testing remains the most accurate approach, we decided to conduct a submaximal bicycle ergometer test, as we are testing older individuals. Undergoing a maximum exercise test could elevate the likelihood of adverse cardiac events (Arena et al., 2007; Sartor et al., 2013) and results are more prone to be biased by musculoskeletal impairments or motivation of a participant (Noonan and Dean, 2000).

The second test to estimate VO_2_max is the 6-min walking test, in which participants have to walk as fast and far as they can on a specified 20-meter route (Mänttäri et al., 2018). Heart rate is constantly measured, and distance is tracked. After the 6 mins, participants are instructed to rest, and blood samples as well as subjective exhaustion scores are taken one and 3 mins after the trial ends.

At last, a non-exercise-based prediction model is used, specifying sports habits, anthropometric measures, and resting sitting heart rate to estimate a person’s VO_2_peak (Nes et al., 2011).

As the study protocol was developed at a time when potential changes in COVID-19 regulations had to be taken into account, we are using three different methods to estimate CRF in terms of VO_2_max and VO_2_peak, respectively. Both the non-exercise-based prediction model and the 6-min walking test could be conducted without the need for a laboratory and while keeping the required minimum distance to the participants. In case we are able to conduct all three tests with all participants and the estimates end up differing from each other, we will use the results of the submaximal bicycle ergometer test because it allows the most accurate estimation of VO_2_max.

After all tests estimating CRF are finished, participants’ handgrip strength is measured with a calibrated Jamar^®^ Smart digital hand dynamometer (Performance Health, Warrenville, IL), according to Southampton protocol for adult grip strength measurement (Roberts et al., 2011). The measurement is conducted in a standardized sitting position, upper arm close to the upper body, and the elbow flexed at a 90° angle. Maximum isometric strength is measured three times (alternating) for both hands and the highest value attained by each hand as well as the overall maximum is used for further calculation.

### General procedure

**Figure 2:**
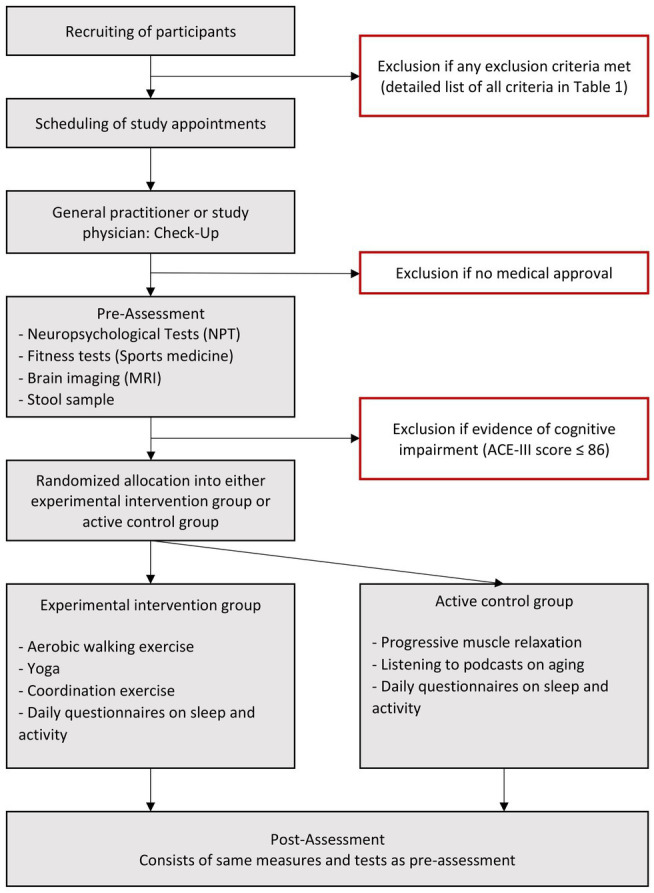
Flow diagram of the study. MRI, Magnetic Resonance Imaging; ACE-III, Addenbrooke’s Cognitive Examination III.

After the successful recruitment of a participant, dates for the pre-assessment are scheduled. The pre-and post-assessment consist of three appointments at three different locations, respectively. Figure 2 shows the procedure flowchart. Before their first appointment, participants receive detailed written information about the study via mail. To preclude the risk of cardiovascular incidents during the self-monitored intervention, all participants have to undergo a checkup examination beforehand. This checkup can be done either by their general practitioner or by the study physician during the first session at the clinic.

The pre-assessment comprises three different sessions. The “NPT session” at JUH, the “Sports session” at the Sports Medicine Department of Friedrich-Schiller University Jena, and the “MRI session” at the JUH research scanner. Every session takes place on a different day and the order in which they are held is not predefined. The only exception is made when participants have a very long drive to Jena. In that case, the MRI session is planned for the same day as the NPT session so that participants only have 2 days on which they have to come to Jena. In general, the three sessions are planned to take place within a maximum of 2 weeks in order to keep the total duration of the study and the time interval between the pre-and the post-assessments as similar as possible between all participants.

In the beginning of the first session, participants have the opportunity to ask questions about the content and procedure of the study. If they agree with all aspects, they sign the informed consent. Also, as described above, a stool collection tube is handed out to each participant.

In the “NPT session,” the majority of the neuropsychological data is assessed. Tests conducted on that day include the Mars contrast sensitivity test, TVA whole and partial report, Addenbrooke’s Cognitive Examination, Mehrfachwahl-Wortschatz-Intelligenztest, and the Trail-Making-Test A-and-B. Furthermore, participants fill out questionnaires, including Becks Depression Inventory, Big Five Inventory, ECog Performance Status Scale, Hospital Anxiety and Depression Scale, Memory Functioning Questionnaire, and the Gesundheitsfragebogen für Patienten. All tests and questionnaires are always performed in the same order and take around 2 h in total.

The “MRI session” takes place at the JUH research scanner. On the MRI session, participants start by reading and signing the MRI information and consent. After that, they hold a pre-treatment consultation with a radiologist. If the radiologist gives his/her approval, the MRI assessment can be conducted. The MRI session consists of a short scout sequence, a structural brain scan, an fMRI resting state as well as a Diffusion Tensor Imaging sequence. Altogether, it lasts approximately 30 min.

The “Sports session” is completed at the Sports Medicine department of the Friedrich-Schiller University Jena. All sports measures are taken and remaining NPTs (VLMT, ROCF, FWIT) are conducted as described above. Furthermore, participants are instructed on how to conduct the intervention. All participants receive a code that grants access to the study website to their respective intervention group contents. The contents as well as general guidelines on how to conduct the intervention are explained. Lastly, the pulse watch (Garmin vivosmart 4) is handed to the participants and its functioning and handling are explained and tested. With the sports measures and tests, the NPTs, and the introduction to the intervention combined, this session takes between two and two and a half hours.

**Figure 3:**
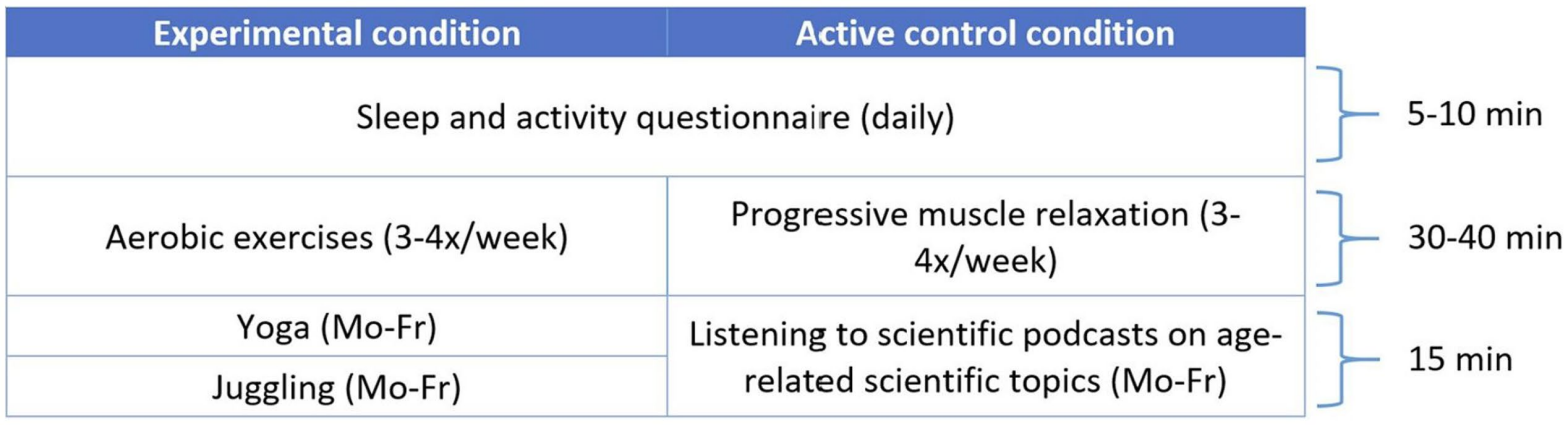
Exercise units and durations of both intervention groups. This Figure shows the timetable of a week during the intervention. Sleep and activity questionnaires are filled out by both groups on a daily basis. In the experimental condition, participants will have aerobic exercise units 3-4 times per week, while participants in the control group do progressive muscle relaxation. During weekdays (Monday to Friday), participants in the experimental condition, will do Yoga and Juggling exercises, while participants in the control group listen to chapters of a scientific podcast on age-related topics.

After finishing the pre-assessment, participants start with their intervention on Monday of the following week. All supplementary materials needed for the conduction (weekly unit overview, questionnaire links, instructions, videos, etc.) can be found on the study website. Both intervention groups are identical concerning their weekly expenditure of time. In the experimental intervention group, participants follow a multicomponent physical activity plan consisting of aerobic exercises (medium-intensity and medium-to-high-intensity walking units), balancing exercises (yoga), and coordination exercises (juggling). A detailed listing of all units and durations can be found in Figure 3. Every walking unit has a specific intensity rating, ranging from “1” (very low intensity) to “4” (high-intensity). For every intensity score, participants find advice on the study website on how to walk in this intensity as well as values from the Borg’s scale of perceived exertion, which they already know from the Sports session of the pre-assessment. The yoga lessons have been designed and recorded specifically for this study. While they can be assumed to entail aerobic parts, they focus on balancing and flexibility improvement for elderly. The aim of the experimental intervention is to increase maximum oxygen uptake in a wholesome and healthy way, as recommended by the World Health Organization and the American College of Sports Medicine (World Health Organization, 2007; Lee et al., 2017). In the active control group, participants listen to podcasts on different age-related scientific topics and perform progressive muscle relaxation exercises, which both have been recorded specifically for this study, at the medical department of the JUH and the sports department of the FSU, respectively. This control group is designed to let participants have the same time expenditure with their units as the experimental intervention group, without influencing their maximum oxygen uptake. Lastly, participants in both groups fill out a short questionnaire about sleep and activity behavior on a daily basis. All participants are instructed to do the units of their respective training plan on top of their existing sports habits so that baseline activity is maintained.

To monitor adherence to the training plan, participants wear pulse watches (Garmin vivosmart 4) that they have to activate during the execution of their units. All recorded data are then synchronized with their individual study Garmin Connect account. This pseudonymized study account is created and set up by the study team and is connected to the mobile phone of the participant during the pre-assessment. Participants are informed and agree that all recorded activities can be read out by the study team. In case too many recordings missing, the study team contacts the respective participant to see if there are technical issues or other problems with the execution of the intervention.

After the intervention phase, the post-assessments take place. In general, the post-assessments comprise the same tests and measures as the pre-assessments, except for the omission of the ACE-III and the MWT-B.

After completion of the post-assessment, participants receive feedback about individual measures from the study. These include a summary of their results from the sports assessment, sleep and exercise data from the daily questionnaires, as well as feedback on whether the results of the cognitive screening test and the MRI were age-appropriate. All these information are sent to the participants in a PDF document via mail and are edited in graphs to be easily comprehensible. If gross pathologies are detected in the MRI or the score in the ACE-III falls below the cut-off of 87 points, participants are informed even before the end of the study.

### Data storage and management

All participant-related data are collected and assigned a pseudonymized study ID. It is not possible to draw any conclusion about the identity of the participant solely from the pseudonym. Only the principal investigator keeps a password-protected list in which pseudonyms are matched with participants’ real names and basic demographic information. This list is stored in the test center and will be saved for 10 years after the end of the study.

For all cognitive and psychological tests and questionnaires collected in the NPT lab of JUH in paper-and-pencil form or on the computer, only the study ID is used, and all data are instantly stored on the internal Hospital server and are password-protected.

MRI scans are done at the research MRI facility of JUH/Friedrich Schiller University. All data collected are stored on the clinical DICOM server and are only accessible with permissions.

Gut microbiome data are collected at participants’ homes. All tubes are labeled with the study ID of the respective participant. The samples are stored by the Integrated Biobank Jena (IBBJ) of the JUH in accordance with their rules of use and with the approval of the relevant ethics committee. Analysis of the stool samples will be done within the ITN SmartAge Consortium.^1^ For that purpose, all samples will be sent to Kiel University, Germany. The stool sample tubes will arrive in Kiel only labeled with participants’ study IDs.

During the eight-week intervention, participants wear pulse watches (Garmin vivosmart 4) and activate them during the execution of certain units. All recorded data are then synchronized with the Garmin Connect App, in which all participants are logged in with their respective study accounts. This account includes no personal data of the participants. When using the pulse watch for the walking units, participants can decide whether or not to use GPS while recording their activity. By analyzing activities for which GPS was used, it would be possible to draw conclusions about the area of residency of a participant. Therefore, all participants are informed beforehand and instructed on how to avoid GPS localization if desired.

### Data analysis plan

For the confirmatory analysis, we plan to test the primary outcome for group differences. To test the hypothesis that the specific intervention group has a higher impact on the primary outcome than the active control group, we will apply an ANCOVA within the framework of general linear models in which the group effect is tested on a two-sided level of significance of α = 0.05, with sex as the stratum and baseline VPS *C* as a covariate.

All statistical analyzes of the secondary outcomes will be carried out in an exploratory manner. All remaining cognitive measures will be analyzed in the same way as the primary outcome.

For the brain measures, we are going to investigate and compare the brain plasticity changes using structural MRI images. For this purpose, we will first implement a voxel based morphometry analysis to look for possible brain structural differences (total gray matter and white matter volume and region based). Then, after calculating the BrainAGE score using a machine learning framework and brain volumes, we will investigate any changes in individual BrainAGE score and brain plasticity induced by physical activity. We will furthermore investigate and compare potential changes in functional and structural brain connectivity within and across large scale brain networks. Functional connectivity will be analyzed by looking at changes in intrinsic resting state networks, and structural connectivity by analyzing changes in white matter fasciculi by using Diffusion Tensor Imaging (DTI).

The stool samples will be processed at Kiel University to perform 16S gene sequencing in order to assess the microbial composition of patients’ feces. Alpha and beta diversity analyzes, as well as constraint-based metabolic models of each sample’s microbial community, will be conducted. If any of the mentioned analyzes will show an association with the primary or a secondary outcome of this study, shotgun metagenomics, as well as metabolomics will be performed on a subset of the samples to increase the resolution of both bacterial composition and metabolic functional content. This will also allow validation of the modeling results and provide evidence for host or bacterial metabolites involved in the gut-brain metabolic cross-talk.

The exploration of the associations between cognitive, brain, and gut-microbiome data will be done using multiple linear regression analyzes. The cognitive measures of the post-assessment (primary outcome: VPS *C*) serve as dependent variables. The respective cognitive measures of the pre-assessment, sex, age, and IQ will be used as covariates. Changes in brain and gut-microbiome measures serve as independent predictors.

### Methodological challenges

With the data collection starting in March 2022, COVID-19 regulations in Germany had to be taken into account. The future situation and development of the pandemic are not foreseeable. Therefore, data collection and the intervention had to be planned accordingly. Instead of in-person group sessions for the intervention units, we decided to lay this out as an online intervention. The participants can execute all of the units themselves without any contact with other participants. This way, changing contact regulations due to governmental decisions will not affect the feasibility of the intervention. All pre-and post-assessments could be conducted with respect to necessary COVID-19 hygiene measures. To minimize the risk of any participant being infected during these assessments, only participants vaccinated against COVID-19 are recruited.

Another potential risk factor arises with participants doing physical exercises without supervision. To decrease the chance of any cardiovascular incident, a physician checks every participant before starting the intervention. After that, participants are assessed concerning their physical fitness and individually instructed on how to conduct the intervention. In case of any problems during the exercises, the study team is always approachable and can provide individual advice based on communication with the Sports Medicine department.

## Ethics statement

This study was approved by the ethics committee of the Faculty of Medicine of Friedrich-Schiller University Jena. The studies involving humans were approved by ethics committee of the Faculty of Medicine of Friedrich-Schiller University Jena. The studies were conducted in accordance with the local legislation and institutional requirements. The participants provided their written informed consent to participate in this study.

## Author contributions

SS: Conceptualization, Investigation, Writing – original draft. SB: Conceptualization, Funding acquisition, Supervision, Writing – original draft. SF: Writing – original draft. CF: Conceptualization, Funding acquisition, Writing – review & editing. CG: Conceptualization, Funding acquisition, Writing – review & editing. RH: Investigation, Writing – review & editing. MH: Conceptualization, Investigation, Writing – original draft. CK: Conceptualization, Funding acquisition, Writing – review & editing. FK: Investigation, Writing – review & editing. H-JM: Investigation, Writing – review & editing. CP: Conceptualization, Writing – review & editing. MR: Investigation, Writing – review & editing. AR-R: Conceptualization, Writing – review & editing. TaS: Conceptualization, Writing – review & editing. AS: Writing – original draft, Conceptualization. ThS: Writing – review & editing. OW: Conceptualization, Funding acquisition, Supervision, Writing – review & editing. KF: Conceptualization, Funding acquisition, Project administration, Supervision, Writing – original draft.

## Funding

This project has received funding from the European Union’s Horizon 2020 research and innovation program under the Marie Skłodowska-Curie grant agreement no 859890. KF received funding from the German Research Foundation (DFG; FI 1424/2-2).

## Conflict of interest

The authors declare that the research was conducted in the absence of any commercial or financial relationships that could be construed as a potential conflict of interest.

## Publisher’s note

All claims expressed in this article are solely those of the authors and do not necessarily represent those of their affiliated organizations, or those of the publisher, the editors and the reviewers. Any product that may be evaluated in this article, or claim that may be made by its manufacturer, is not guaranteed or endorsed by the publisher.

## Footnotes

^1^https://www.uniklinikum-jena.de/etnsmartage/en/Consortium.html.
